# *In vitro* antifungal activity of pelgipeptins against human pathogenic fungi and *Candida albicans* biofilms

**DOI:** 10.3934/microbiol.2021003

**Published:** 2021-01-19

**Authors:** Débora Luíza Albano Fulgêncio, Rosiane Andrade da Costa, Fernanda Guilhelmelli, Calliandra Maria de Souza Silva, Daniel Barros Ortega, Thiago Fellipe de Araujo, Philippe Spezia Silva, Ildinete Silva-Pereira, Patrícia Albuquerque, Cristine Chaves Barreto

**Affiliations:** 1 Graduate Program in Genomic Sciences and Biotechnology, Catholic University of Brasília, Brasília, Brazil; 2 Laboratory of Molecular Biology, Department of Cellular Biology, Institute of Biological Sciences, University of Brasília, Brasília, Brazil; 3 Faculty of Ceilandia, University of Brasília, Brasília, Brazil

**Keywords:** lipopeptides, systemic mycosis, *Candida* spp, *Cryptococcus neoformans*, *Paracoccidioides brasiliensis*, *Paenibacillus elgii*

## Abstract

Systemic mycoses have become a major cause of morbidity and mortality, particularly among immunocompromised hosts and long-term hospitalized patients. Conventional antifungal agents are limited because of not only their costs and toxicity but also the rise of resistant strains. Lipopeptides from *Paenibacillus* species exhibit antimicrobial activity against a wide range of human and plant bacterial pathogens. However, the antifungal potential of these compounds against important human pathogens has not yet been fully evaluated, except for *Candida albicans*. *Paenibacillus elgii* produces a family of lipopeptides named pelgipeptins, which are synthesized by a non-ribosomal pathway, such as polymyxin. The present study aimed to evaluate the activity of pelgipeptins produced by *P. elgii* AC13 against *Cryptococcus neoformans*, *Paracoccidioides brasiliensis*, and *Candida* spp. Pelgipeptins were purified from *P. elgii* AC13 cultures and characterized by high-performance liquid chromatography (HPLC) and mass spectrometry (MALDI-TOF MS). The *in vitro* antifugal activity of pelgipeptins was evaluated against *C. neoformans* H99, *P. brasiliensis* PB18, *C. albicans* SC 5314, *Candida glabrata* ATCC 90030, and *C. albicans* biofilms. Furthermore, the minimal inhibitory concentration (MIC) was determined according to the CLSI microdilution method. Fluconazole and amphotericin B were also used as a positive control. Pelgipeptins A to D inhibited the formation and development of *C. albicans* biofilms and presented activity against all tested microorganisms. The minimum inhibitory concentration values ranged from 4 to 64 µg/mL, which are in the same range as fluconazole MICs. These results highlight the potential of pelgipeptins not only as antimicrobials against pathogenic fungi that cause systemic mycoses but also as coating agents to prevent biofilm formation on medical devices.

## 1. Introduction

An increasing incidence of systemic mycoses has been observed in the last three decades, particularly in immunocompromised individuals, including patients with AIDS and/or cancer, submitted to organ transplantation, undergoing corticosteroid therapy, or in intensive care units [1]–[3].

*Candida* infections have increased not only in occurrence but also in severity, and invasive candidiasis is a major cause of health-care-associated morbidity and mortality, especially in older adults and newborns [4]. They are part of the human and animal microbiota, and most of the infections have an endogenous origin. The balance between this microorganism and its host depends on yeast virulence and host-related factors, such as comorbidities, immunity, and age [1],[4],[5]. *Candida* is also the most common yeast genus to cause infections by biofilm formation because it can colonize internal and external medical devices, giving rise to adherent biofilms. The release of yeast cells from biofilms can lead to generalized infection or fungemia [6].

*Cryptococcus neoformans*, one of the etiological agents of cryptococcosis, is an encapsulated yeast that lives primarily in the environment and is frequently associated with bird excreta or trees. Meningitis or meningoencephalitis caused by *C. neoformans* is a major cause of fungal-related death in patients with AIDS [7]–[10].

Paracoccidioidomycosis is a systemic neglected mycosis endemic in Latin America. The disease is caused by dimorphic fungus *Paracoccidioides* spp, which is found as a mycelium at an environment temperature of 25 °C and as yeast in host tissues at 37 °C [11]–[13]. Moreover, the disease primarily affects poor male rural workers at their most productive age, and it is a crucial cause of work disability during an active disease or even after it due to disease sequelae [14].

Despite the increase in the incidence and relevance of fungal infections, the number of available antifungals is extremely limited. The fact that fungi and animals are phylogenetically close makes the search for antifungal targets with high selective toxicity a difficult task. Most of the systemic antifungals currently in use are toxic to mammalian cells, and their costs are high; antifungal resistance is emerging in several pathogenic fungi. To address these problems, several groups have been working in the identification of new antifungal molecules and in the development of new therapeutic approaches to improve the treatment of systemic fungal infections.

Bacterial lipopeptides are a class of antimicrobial peptides (AMPs) with activity against a wide range of pathogens. These molecules are assembled following a non-ribosomal pathway by a large multi-enzyme complex named non-ribosomal peptide synthetases (NRPSs). Lipopeptides are usually small cyclic or linear oligopeptides associated with either a lipid tail or a lipophilic molecule and also contain non-proteinogenic amino acids [15],[16]. *Bacillus* and *Paenibacillus* species produce several antimicrobial lipopeptides and are commonly employed as biocontrol agents against plant pathogens [17],[18]. Hitherto, the clinical use of lipopeptides has been restricted due to their toxicity to human cells, which is also exhibited by some compounds, such as polymyxins [16]. However, the emergence of multidrug-resistant pathogens has increased the number of studies on safer and more efficient antimicrobial lipopeptides [19].

Pelgipeptins are cyclic lipopeptides produced by *Paenibacillus elgii* that present antimicrobial activity against several bacterial pathogens [20]–[22]. Nonetheless, their activity against clinically important fungi has not yet been evaluated, except for *Candida albicans*. These cationic peptides originated from non-ribosomal synthesis comprising four well-known homologous compounds: pelgipeptins A, B, C, and D (hereafter named isoforms) [20],[21]. In the present work, we evaluated the *in vitro* antifungal activity of pelgipeptins against the planktonic cells of the three genera of human pathogenic fungi and their potential to inhibit *C. albicans* biofilm formation.

## 2. Materials and methods

### 2.1. Strains and growth conditions

*Paenibacillus elgii* AC13 was isolated from Brazilian Cerrado soil [23], and it produced four pelgipeptin isoforms as found in *P. elgii* B69 [20],[21] (Table 1). The purified spores of *P. elgii* AC13 were inoculated in 250 mL flasks containing 100 mL of MMP medium (pH 7.0) (Patent BR102017018881 7) at a final concentration of 10^3^ spores/mL and incubated at 37 °C, 200 rpm, for 72 hours.

**Table 1.** Amino acid sequence and fatty acid composition of the pelgipeptin isoforms produced by *Paenibacillus elgii* AC13.

Pelgipeptin isoform	[M + H]^+^ m/z	Fatty acid	Amino acid sequence	Reference
A	1073	C_6_H_10_O_2_	Dab1-Val2-Dab3-Phe4-Leu5-Dab6-Val7-Leu8-Ser9	[20],[22]
B	1101	C_7_H_12_O_2_	Dab1-Ile2-Dab3-Phe4-Leu5-Dab6-Val7-Leu8-Ser9	[20],[22]
C	1087	C_7_H_12_O_2_	Dab1-Val2-Dab3-Phe4-Leu5-Dab6-Val7-Leu8-Ser9	[21],[22]
D	1087	C_6_H_10_O_2_	Dab1-Ile2-Dab3-Phe4-Leu5-Dab6-Val7-Leu8-Ser9	[21],[22]

Dab: 2,4-diaminobutyric acid.

The isolated pelgipeptin isoforms were tested against the reference clinical strains of *Candida albicans* SC 5314 (ATCC MYA-2876), *Candida glabrata* ATCC 90030, *Cryptococcus neoformans* H99 (ATCC 208821), and *Paracoccidioides brasiliensis* PB18, a Brazilian clinical isolate. All the isolates were obtained from the mycology collection of the University of Brasilia (Department of Cell Biology). Before each experiment, both *Candida* spp. strains were grown in Sabouraud's dextrose broth (Himedia) overnight at 30 °C/200 rpm. In addition, the *C*. *neoformans* cells were grown in the same conditions for 24 h. *P. brasiliensis* strains were also grown in GPY medium (2% glucose w/v, 1% peptone w/v, 0.5% yeast extract w/v, and 2% agar w/v), at 37 °C, for 5 days. The fungal cells were collected by centrifugation, washed thrice in phosphate buffered saline (PBS) (NaCl, 137 mM; KCl, 2.7 mM; Na2HPO4, 10 mM; KH2PO4, 2 mM), and inoculated in RPMI 1640 medium (Thermo-Fisher Scientific) supplemented with L-glutamine and buffered to pH 7.0 with 165 mM of 3-(N-morpholino) propanesulfonic acid.

### 2.2. Pelgipeptin purification and analysis

*P. elgii*'s AC13 culture was centrifuged at 9000 *× g* for 10 minutes. The cell free supernatant was filtered using a 0.22 µm membrane and purified by solid-phase extraction (SPE) using a syringe-cartridge Strata XL 100 µm Phenomenex®. The cartridge was also washed with acetonitrile (ACN) acidified with trifluoracetic acid (TFA) 0.1% at concentrations of 5, 10, 20, 30, 40, 60, and 90% ACN. The pelgipeptin mixture containing all the four pelgipeptin isoforms (pelgipeptin mix) was then obtained from the 40% ACN elution.

The pelgipeptin mix was purified by high-performance liquid chromatography (HPLC) (Shimadzu, Japan) using a reverse phase column Shimadzu C18 Shim-pack VP-ODS (4.6 µm, 150 × 4.6 mm) to obtain the isolated isoforms. Mobile phase A was HPLC-grade water containing 0.1% of TFA, and mobile phase B was acetonitrile and 0.1% of TFA. The elution was monitored at 216 nm utilizing a UV detector (Prominence, Shimadzu, Japan). Additionally, the fractions were collected manually, and the lipopeptide identities were confirmed by mass spectrometry (MALDI-TOF MS) (Autoflex Speed; Bruker Daltonics, Germany) on reflected-positive mode (700–3500 m/z). Thereafter, the fractions of the same retention time that originated from multiple runs were pooled and re-chromatographed, following the parameters described above.

The pelgipeptin stock solutions were quantified by the Murphy and Kies method using UV absorption at 205, 215, and 225 nm [24]. The isolated isoforms and the pelgipeptin mix were used in the antifungal experiments.

### 2.3. Antifunal assay

The *in vitro* antifungal assays were performed according to the broth microdilution susceptibility test from the Clinical and Laboratory Standards Institute M27-A4 guidelines [25] with modifications [26]. Briefly, the twofold serial dilutions of each pelgipeptin were prepared in 96-well polystyrene microplates to a final volume of 50 µL. Fluconazole, an antifungal employed in the treatment of mycosis caused by *Candida* spp, *Cryptococcus* spp, and *Paracoccidioides* spp, was used as a control (Sigma-Aldrich). We also utilized polymyxin B (Sigma-Aldrich), another bacterial lipopeptide, in our assays due to some works that show its antifungal activity [27],[28]. The final concentrations of each lipopeptide and control ranged from 1 to 128 µg/mL, and the negative controls without peptide were used to assess fungal growth. Subsequently, 50 µL of the inoculum adjusted on RPMI-1640 medium was added to each well to a final concentration of 2 × 10^3^ cells/mL for *Candida* spp. and 10^4^ cells/mL for *C. neoformans* or *P. brasiliensis* strains. The microplates were incubated at 37 °C for 24, 48, or 72 h. In addition, the strains of *C. neoformans* were incubated with shaking at 200 rpm.

The minimum inhibitory concentration (MIC) was defined as the lowest concentration that completely inhibited visible fungal growth at the end of the incubation period. The MICs of the pelgipeptins against *P. brasiliensis* were determined using the microplate AlamarBlue Cell Viability Assay [29]. This assay uses a resazurin-based solution to indicate cell viability, which was evaluated by fluorometric readings in a fluorescence plate reader with excitation at 550 nm and emission at 585 nm (SpectraMax® M plate reader). The experiments were performed in triplicates at least thrice on separate dates.

### 2.4. Effects of pelgipeptins on Candida albicans biofilms

The effects of the pelgipeptins on *C. albicans* biofilms were analyzed according to the protocol described by Pierce et al. [30]. Briefly, 2 × 10^6^ cells/mL of *C. albicans* SC 5314 were inoculated on 96-well polystyrene microplates. The inhibition of biofilm formation was also evaluated by adding antimicrobials (lipopeptides, fluconazole, or amphotericin B) at the same time as the yeast inoculum. Furthermore, the inhibition of early-phase biofilms was assessed after 4 hours of yeast growth by removing the planktonic cells and adding the antimicrobials (lipopeptides or amphotericin B). For the investigation of the effects of peptides on mature biofilms, the *Candida* cells were allowed to grow for 24 h when planktonic cells were removed, and the antimicrobials were added to the culture and incubated for another 24 h at 37 °C.

The biofilm viability was analyzed by the metabolic assay on the basis of the reduction of 2,3-bis-(2-methoxy-4-nitro-5-sulfophenyl)-2H-tetrazolium-5-carboxanilide (XTT) and absorbance reading at 490 nm. The final concentrations of each pelgipeptin and pelgipeptin mix ranged from 1 to 128 µg/mL, polymyxin ranged from 8 to 1024 µg/mL, and amphotericin B or fluconazole concentrations ranged from 0.25 to 64.0 µg/mL. Furthermore, each compound was tested in triplicate, and the experiments were performed at least twice on separate dates. The sessile minimal inhibitory concentrations (SMICs) were also determined at 80% (SMIC80) and 50% (SIMIC50) decrease in absorbance at 490 nm in comparison with the control biofilms growing in the absence of the drug, as previously described [30].

## 3. Results

**Figure 1.**
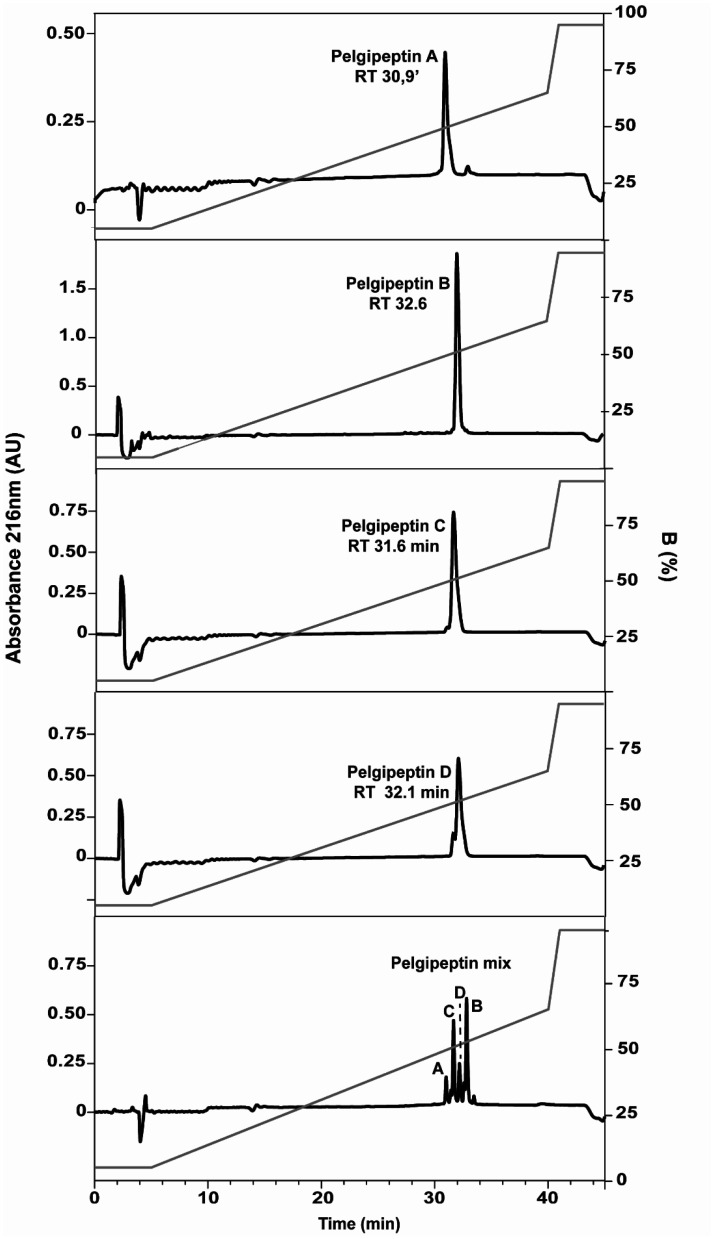
Reverse-phase chromatography of the four pelgipeptins isoforms (A to D) stock solutions and Pelgipeptin mix purified from P. elgii AC13's cell-free supernatant. Optimized gradient is shown as a gray line and detection at 216 nm as black line. The letters correspond to the pelgipeptin isoforms. RT: retention time.

A mixture of pelgipeptins (pelgipeptin mix) was obtained after the solid phase extraction of the cell-free supernatant. This mixture was submitted to liquid chromatography to obtain the isolated pelgipeptin isoforms (Figure 1). The chromatograms of the pelgipeptins and the pelgipeptin mix with their respective retention time showed the level of purity of each pelgipeptin (Figure 1). The antimicrobial assays were also performed with stock solutions where the purity was as follows: pelgipeptin A (94%), pelgipeptin B (99%), pelgipeptin C (98%), and pelgipeptin D (88%). The pelgipeptin mix shows the following pelgipeptin isoform composition: pelgipeptin A (9%), pelgipeptin B (37%), pelgipeptin C (28%) and pelgipeptin D (15%).

**Figure 2.**
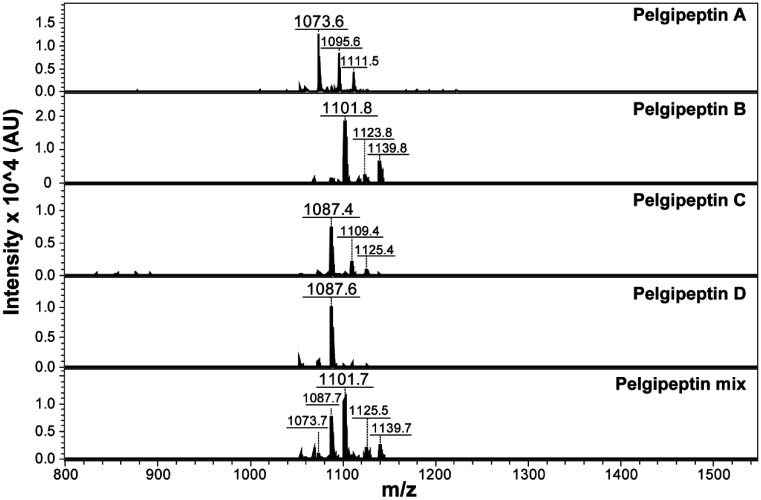
Mass spectra (MALDI-TOF-MS) of pelgipeptin stock solutions. Pelgipeptin A [M + H]^+^: 1073.6; [M + Na]^+^: 1095.6; and [M + K]^+^ 1111.5 m/z. Pelgipeptin B [M + H]^+^: 1101.8; [M + Na]^+^: 1123.8; and [M + K]^+^ 1139.8 m/z. Pelgipeptins C and D [M + H]^+^: 1087; [M + Na]^+^: 1109; and [M + K]^+^ 1125 m/z. The pelgipeptin mixture exhibits all ions corresponding to the four isoforms. [M + Na]^+^: and [M + K]^+^ correspond to sodium and potassium aducts, respectively.

The mass spectra of the purified stock solutions used on the antifugal assays revealed the three expected mass/charge ratios [M+H]^+^ corresponding to pelgipeptin A (1073.6 m/z), pelgipeptin B (1101.8 m/z), pelgipeptin C (1087.4 m/z), and pelgipeptin D (1087.6 m/z), and the pelgipeptin mix (1073, 1087, and 1101 m/z) was detected and purified from the supernatants of *P. elgii* AC13 (Figure 2).

The purified pelgipeptins and the mixture were tested against the reference strains of four clinically relevant fungal species: *C. albicans*, *C. glabrata*, *C. neoformans*, and *P. brasiliensis*. Overall, pelgipeptin B showed the lowest MICs for all the tested fungal strains in comparison with the other pelgipeptins (Table 2). *C. neoformans* was the species most sensitive to all the pelgipeptins, presenting MICs of 4 µg/mL for pelgipeptin B or the pelgipeptin mix and 8 µg/mL for the other pelgipeptins alone and a MIC of 2 µg/mL for fluconazole. Similarly, *C. neoformans* was also the strain most sensitive to polymyxin B in contrast with *P. brasiliensis,* which was able to grow even in concentrations as high as 1024 µg/mL of this lipopeptide. Conversely, *C. glabrata* exhibited the highest MICs for the tested compounds, as previously reported for several other antifungals. Interestingly, the treatment of both *Candida* spp species with pelgipetins or fluconazole resulted in similar MICs (Table 2).

**Table 2.** Minimum inhibitory concentration of antimicrobial lipopeptides produced by *Paenibacillus elgii* AC13 against common human fungal pathogens.

	MIC (µg/mL)			
Lipopeptide	*Paracoccidioides brasiliensis* Pb18	*Cryptococcus neoformans* H99	*Candida glabrata* 90030	*Candida albicans* SC5314
Pelgipeptin A	NT	8	64	16
Pelgipeptin B	8	4	32	8
Pelgipeptin C	32	8	64	16
Pelgipeptin D	NT	8	32	8
Pelgipeptin mix	8	4	32	8

Controls

Fluconazole	2	2	32	16
Polymyxin B	1024	8	512	256

NT: Not tested

All the pelgipeptins were active inhibiting *C. albicans* initial biofilm formation and early-phase biofilms at SMIC_80_ of 16 µg/mL, except for Pelgipeptin D, which inhibited biofilm formation at SMIC_80_ 8 µg/mL (Table 3). As expected, the higher concentrations of pelgipeptins were necessary to inhibit mature biofilms. The effect of amphotericin B was also evaluated for comparison, showing SMIC lower than 0.5 µg/mL for biofilm formation and initial biofilm adhesion. A SMIC50 of 0.5 µg/mL and SMIC80 of 4 µg/mL were observed for mature biofilms (Table 3). Fluconazole did not inhibit biofilm formation in any of the tested concentrations, as previously reported by several groups (Table 3).

**Table 3.** Sessile minimum inhibitory concentration of pelgipeptins at different time points of *Candida albicans* biofilm formation.

	SMIC (µg/mL)		
Lipopeptide	Inhibition of initial biofilm formation SMIC_80_	Inhibition of early-phase biofilm SMIC_80_	Inhibition of mature biofilms SMIC_50_/SMIC_80_
Pelgipeptin A	16	16	32/64
Pelgipeptin B	16	16	32/64
Pelgipeptin C	16	16	64/64
Pelgipeptin D	8	16	>64/64
Pelgipeptin mix	16	16	16/32

Controls

Amphotericin B	<0.5	<0.5	0.5/4
Fluconazole	> 64	NT	NT

NT: Not tested

## 4. Discussion

Lipopeptides produced by bacteria have long been known for their antimicrobial activity. However, their clinical applications were hindered by their toxicity to host cells, and their therapeutic use only became widespread in the 21st century [16],[31]. The emergence of multidrug-resistant pathogens combined with new therapeutic practices overcame initial concerns on their toxicity to human cells and propelled the development of approaches to decrease their toxicity, such as their use in combination with other antimicrobials [16],[32]. For instance, polymyxins are lipopeptides produced by *Paenibacillus polymyxa*, and they are currently used against resistant gram-negative bacteria [32]. Furthermore, they were reported to show activity against filamentous fungi and yeasts [19],[33].

Pelgipeptins produced by *P. elgii* present several similarities to polymyxins. Both groups of compounds comprise cyclic lipopeptides produced by a non-ribosomal pathway, possess 2,4-diaminobutyric acid as a non-proteinogenic amino acid, and present D-amino acids in their primary sequence. Nevertheless, polymyxins are decapeptides, whereas pelgipeptins are nonapeptides; they also differ in their fatty acid composition. Cyclic pelgipeptins also exhibit a lactone ring that is not present in polymyxins [20],[32],[34].

Although both groups of lipopeptides present activity against bacteria [21],[22], our results show that they differ in their antifungal activity. Our bioassays show that pelgipeptins are effective against all pathogenic fungi tested, presenting MICs similar to fluconazole and consistently lower than MICs exhibited by polymyxin B. Reasons for the differences in MIC values between polymyxin B and pelgipeptins are not yet known, but pelgipeptins' low MICs against pathogenic fungi indicate their potential for therapeutic use, especially considering the emergence of fungal resistance to diverse antifungals [35].

The use of a mixture of pelgipeptin could be considered a substitute for the use of purified isoforms because there is a synergic effect between the pelgipeptin isoforms [22]. Moreover, obtaining pelgipeptin mix through a simple extraction in a solid phase, instead of repeated chromatographic runs, is a faster and less expensive alternative. Contrarily, the mixture may present undesirable toxic effects given that each pelgipeptin may present divergent toxicity levels. The pelgipeptin mechanism of action and their cytotoxicity are not yet clear. Previous results on the cytotoxicity of pelgipetin D revealed a hemolytic activity lower than 50% at a concentration of 100 µg/mL [21]. This concentration is higher than the MIC of pelgipeptin D for *C. neoformans* H99, *C. albicans* SC5314 (8 µg/mL), and *C. glabrata* ATCC 90030 (32 µg/mL) observed in the present study. In addition, a new linear isoform of pelgipeptin as isolated from *P. elgii* BC34-6. Pelgipetin E exhibited significant lower hemolytic activity when compared with the membrane-active peptide melittin, a lipopeptide obtained from bee venom. The mode of action of pelgipeptin E against bacteria is likely to be a depolarization of the cell membrane [36]. Biofilm formation increases *C*. *albicans* resistance against fluconazole and amphotericin [37]. This effect is more noticeable in mature biofilms as the addition of amphotericin in the initial steps of biofilm formation inhibits yeast growth [38]. Under our experimental conditions, pelgipeptins showed inhibitory activity in the formation and adhesion of *C. albicans* biofilm and action against mature biofilm. The inhibition concentration values were comparable with the results exhibited by amphotericin B. The biofilm inhibition values by amphotericin B were also close to those found in other works [39],[40]. These results indicate the biotechnological potential of pelgipeptins against *C. albicans* biofilm in several stages of its formation.

## 5. Conclusion

The results suggest that pelgipeptins exhibit the potential to inhibit the growth of pathogenic fungi in humans. In addition, pelgipeptins inhibit the formation, adhesion, and growth of planktonic cells in *C. albicans* mature biofilms. The MIC values were comparable with the ones presented in trials involving fluconazole and amphotericin B, which are two of the most commonly used antifungals. However, until the elucidation of their toxicity and mechanism of action against fungi, its use requires further studies. Other applications can also be considered, such as the use of pelgipeptins as antimicrobial-coating biomaterials against *Candida* biofilms.
